# Kinematics of balance controls in people with chronic ankle instability during unilateral stance on a moving platform

**DOI:** 10.1038/s41598-025-85220-x

**Published:** 2025-01-07

**Authors:** Xiaohan Xu, Joanna Bowtell, Daniel T. P. Fong, William R. Young, Genevieve K. R. Williams

**Affiliations:** 1 Public Health and Sports Sciences Department, University of Exeter, Exeter, EX1 2LU UK; 2 National Centre for Sport and Exercise Medicine, School of Sport, Exercise and Health Sciences, Loughborough University, Loughborough, LE11 3TT UK

**Keywords:** Balance control, Kinematics, Chronic ankle instability, Postural strategy, Perturbation, Principal component analysis, Rehabilitation, Musculoskeletal system, Motor control, Sensorimotor processing, Orthopaedics

## Abstract

Balance control deficits resulting from ankle sprains are central to chronic ankle instability (CAI) and its persistent symptoms. This study aimed to identify differences in balance control between individuals with CAI and healthy controls (HC) using challenging single-leg balance tasks. Twenty-three CAI and 23 HC participants performed balance tasks on a force plate that either remained static or moved mediolaterally. Force and kinematic data were recorded to measure balance and joint movements. The CAI group showed significantly shorter time-to-boundary during static conditions but no significant differences during moving conditions compared to HC. During moving conditions, CAIs exhibited greater proximal compensations, with greater range of motion and higher angular velocity in the knee, hip, and torso. while no significant differences were observed in these parameters during static conditions. Principal component analysis indicated specific kinetic chain in CAI during one-leg balance under both static and moving conditions compared to HC. These findings suggest an altered movement strategy in CAI, that ankle injuries impair the ability to stabilize both distal and proximal joints, and an altered kinetic chain from ankle to torso. Rehabilitation programs for CAI might benefit from considering the integration of the entire kinetic chain, addressing both distal and proximal joint dynamics to support effective recovery and prevent secondary injuries.

**Supplementary Information:**

The online version contains supplementary material available at 10.1038/s41598-025-85220-x.

## Introduction

Lateral ankle sprains remain the most prevalent musculoskeletal injury in both athletes and the general population^1^. Epidemiological studies have highlighted the high prevalence rates, with an estimated 70% of the general population experiencing an ankle sprain at some point in their lives^2^. These injuries not only cause immediate functional limitations but also have long-term consequences, since about 50% of people with ankle sprains develop chronic ankle instability (CAI)^3^. CAI is characterised by a persistent state of mechanical and functional instability, affecting postural control and increasing the risk of further injuries^4^.

Postural control deficits are common in individuals with CAI, impacting their ability to maintain balance^5^. Specifically, CAI individuals exhibit increased joint laxity, and decreased neuromuscular control, which compromises their stability and postural control^4^. Research focusing on unilateral weight-bearing tasks, such as single-leg stance^6^, landing^7,8^, and lateral stepping down^9,10^, has demonstrated significant alterations in postural control as well as lower limb kinematics among individuals with CAI.

In double-leg standing balance, postural control strategies are well-defined in the sagittal plane, involving coordinated actions of the ankle, knee, and hip. For instance, Taleshi et al. (2022)^11^ have demonstrated that these joints work together to stabilise the body during forward and backward movements. However, understanding balance control in the frontal plane and under dynamic conditions remains challenging. For example, whole-body contributions to balance control in the mediolateral direction have been observed^12^, which poses the question of the relevant joints involved (i.e. exclusively lower limb, or whole body). In CAI populations, recent studies have identified movement adaptations that shift reliance from ankle strategies to hip strategies in the sagittal plane, involving proximal kinematic and kinetic changes at the knee and hip^4^. Besides, it has been reported that individuals with CAI have greater rates of low back pain and trunk muscular impairment. For instance, Nadler et al. (1998)^13^ found higher incidences of low back pain in CAI individuals, while Marshall et al. (2009)^14^ and Terada et al. (2016)^15^ documented trunk muscular impairments. McCann et al. (2021)^16^ also noted that CAI affects transversus abdominis function, which can compromise feedforward motor control when anticipating an external perturbation or preparing movement. Previous studies on CAI might have overlooked the importance of the trunk for balance, focusing instead primarily on the lower limbs. It is reasonable to suspect that movements of the torso, which shift the body’s centre of mass, would also affect postural control, especially in challenging tasks. To address this, a comprehensive analysis of whole-body kinematics is warranted.

Principal component analysis (PCA) is a multivariate statistical method that has been widely utilised in biomechanical research^17–20^. PCA reduces the complexity of kinematics data, enabling researchers to focus on the most significant components that distinguish movement characteristics across different groups and conditions without requiring prior selection of key features (e.g. peaks)^21^. For example, Promsri et al. (2020)^22^ used PCA to identify leg dominance effects during challenging balance exercises, and Kobayashi et al. (2014)^19^ used PCA to differentiate the walking pattern between fallers and non-fallers. Previous studies identified kinematic alterations in proximal joints^23^, but lacked a full analysis of the entire kinetic chain, leaving the interconnected nature of these alterations unclear. Any blockage or defect in the kinetic chain can lead to compensatory patterns, overuse, and overload injuries, highlighting the importance of rehabilitation strategies that target both the injured area and the functional integration of the entire kinetic chain to support recovery and prevent secondary injuries^24^. In this study we aim to use PCA for comprehensive understanding of ankle-to-torso variations during a challenging balance task, distinguishing movement patterns in individuals with CAI from those of healthy controls.

Postural control is most commonly quantified by the magnitude and velocity of centre of pressure (COP) excursions, while time-to-boundary (TTB) has been argued to be a more sensitive measure in determining postural control deficits than traditional COP measures in CAI^9^. Nevertheless, equivocal evidence exists for the balance deficits in individuals with CAI reported by a meta-analysis^25^. By bridging 3D kinematics of whole-body movement with COP/TTB assessments of stability, it is possible to comprehensively evaluate postural control in relation to specific movement techniques.

Given the high heterogeneity in results of postural deficits among CAI individuals reported in a meta-analysis^25^, we aim to apply a challenging balance task to highlight the postural deficits. In our study, we introduced a moving base of support perturbation in the mediolateral direction, requiring participants to perform whole-body movements, primarily involving the hip and torso, to maintain balance when their stability was challenged. This sinusoidal perturbation challenges both agonist and antagonist muscles, necessitating increased co-contractions to effectively manage movements in multiple directions.

The primary aim of this study was to identify differences in balance between subjects with CAI and healthy control (HC) under two balance conditions (i.e. moving and static platform). We hypothesized that CAI would demonstrate deficits in TTB measures of balance control. To further understand the balance mechanisms in CAI, the second aim was to explore differences in balance strategy using PCA and discrete joint variables. Previous research has reported alterations in kinematics in CAI individuals^9^. We, therefore, hypothesized that the key joint position patterns would differ between groups, and further that balance control in TTB measures would be significantly predicted by joint angular velocities in the sagittal, frontal, and horizontal planes at the ankle, knee, hip, and torso.

## Method

### Participants

A total of 23 participants with CAI and 23 HC were recruited for this study, based on an a priori power analysis requiring at least 23 per group to detect significant differences with an effect size over 0.40^26^, 85% power, and a 5% alpha level by a 2 × 2 repeated between-factor ANOVA. Participants were instructed about the study purpose and procedures and signed written informed consent prior to participating. The University Faculty Sports and Health Sciences Ethics Committee approved the study (1071581). All methods were performed in accordance with the relevant guidelines and regulations.

Both groups were similar in gender, age, height, body mass and physical activity level assessed by the International Physical Activity Questionnaire-Short Form (IPAQ-SF) (Table 1). The CAI group selection followed the criteria of the International Ankle Consortium^27^ and the Cumberland Ankle Instability Tool < 24 was used to identify impaired ankle function^28^. Inclusion criteria for the control group was being free from a history of lateral ankle sprains. Both groups were required to be between the ages of 18 and 35 years old and be free from acute musculoskeletal injury in the 3 months prior to testing, free from visual, hearing disorders, dizziness, recurrent falls, vestibular dysfunction, previous fractures, pain and surgery within the lower extremity, and had no experience in professional balance training.

**Table 1:** Participants’ demographics and anthropometrics.

	CAI (*n* = 23)	HC (*n* = 23)
Gender	Female − 9	Female − 10
	Male − 14	Male − 13
Age, years - mean ± SD	24.7 ± 4.3	24.8 ± 4.0
Height, m - mean ± SD	1.72 ± 0.07	1.73 ± 0.07
Body Mass, kg - mean ± SD	72.9 ± 10.7	67.4 ± 7.6
IPAQ-SF	Moderate − 12	Moderate − 12
	High − 11	High − 11
CAIT score	17.3 ± 3.5	30.0 ± 0

SD: standard deviation; IPAQ-SF: International Physical Activity Questionnaire-Short Form CAIT: Cumberland Ankle Instability Tool.

### Procedure

**Fig. 1:**
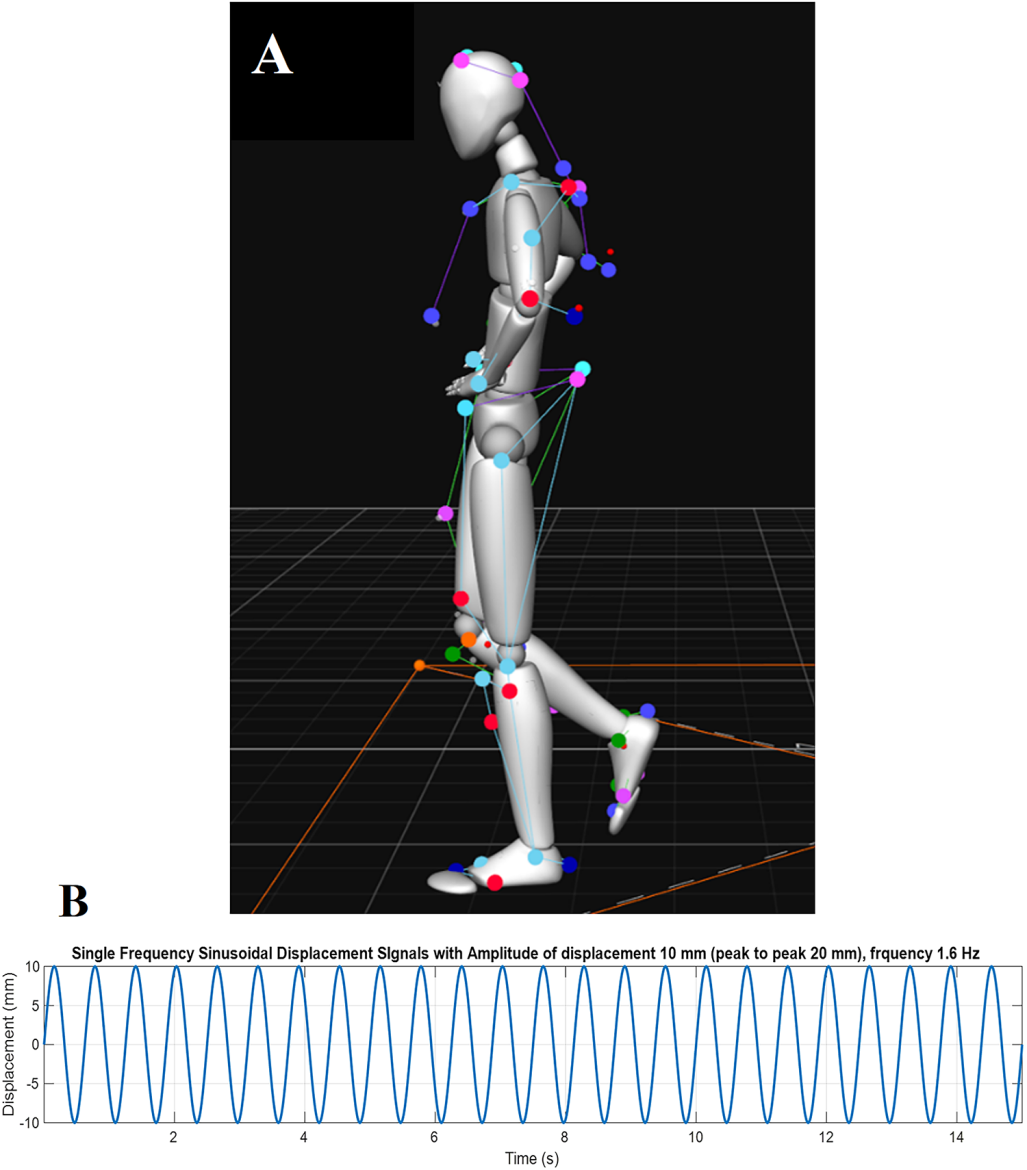
**A** Standardised single-leg standing visualised through markers by motion capture and Motive software (version 2.1.1; https://optitrack.com/software/motive/). **B** Platform motion in 15-s: mediolateral sinusoidal at 1.6 Hz, with displacement (peak-to-peak displacement at 20 mm).

Participants were required to perform single-leg balance tests barefoot (Fig. 1A), on the affected side in CAI. For participants with a history of bilateral ankle sprains, the more affected side, determined by the lower CAIT score, was tested. Height-, gender-, and IPAQ-SF-matched HCs were tested on the corresponding side, and we assumed a similar distribution of leg dominance across both groups. While leg dominance may influence balance strategies, a recent review suggests the effect is not significant^29^. Each balance trial was maintained for a minimum of 15 s and repeated three times. In case of failure, the task was repeated until three complete measures were obtained. The balance was tested on a static and moving platform. The platform’s motion parameters were determined through piloting to create a challenging yet safe and precisely reproducible balance perturbation. The platform motion was produced in the mediolateral direction designed to elicit ankle inversion/eversion responses relevant to CAI. We decided on a sinusoidal motion of the platform in order to challenge balance based on two changes in direction of the platform. Unlike traditional balance tasks with uncontrolled variability, such as balancing on foam, the moving platform provided a consistent and controlled challenge, enabling precise comparisons of responses. The platform’s peak acceleration was limited to 1 m/s² to ensure participant safety, as higher accelerations increase joint reaction forces^11^ and recovery demands^30^. Through piloting, we determined that a 1-cm amplitude (10% of the base of support in ML, assuming an average foot width 10-cm) provided an optimal challenge for participants, allowing them to maintain a single-leg stance for 15 s while eliciting meaningful recovery efforts, though multiple attempts were required in some cases. The final perturbation followed a mediolateral sinusoidal pattern at 1.6 Hz with a peak-to-peak displacement amplitude of 2 cm (Fig. 1B). Participants were asked to keep the standing foot pointed straight ahead. The non-supporting leg was elevated, maintaining approximately 45° knee flexion and 20° hip flexion, without internal or external rotation. Hands were placed on the waist, involving 20–30° of shoulder abduction primarily at the glenohumeral joint^31^, with minimal scapulothoracic involvement, making any impact on torso angles negligible. The lifted leg was kept from touching the standing leg, ensuring the distance between the knees was consistent with pelvic width. Participants were directed to focus their gaze on a target positioned at eye level, approximately three meters away on a wall. Participants were asked to avoid any unnecessary movements (e.g., scratching), during balance to maintain consistency. An unsuccessful trial was defined as any instance of losing balance, the standing foot taking additional steps, making jumps, or creating frictional movements against the platform surface, as well as visually apparent movements of the lifted legs and arms beyond minor adjustments. The successful 15-second trials were trimmed to extract the middle 10 s, starting at the zero-crossing point where the platform began moving medially. This 10-second segment, corresponding to 16 cycles of perturbation at 1.6 Hz, was analysed.

### Data collection and analysis

Participants stood on a 3.6 m by 3.6 m moveable fully instrumented floor in the Exeter VSimulators facility^11^. Three-dimensional positional data was obtained using reflective markers and a 3D motion capture system (OptiTrack, NaturalPoint Inc., Corvallis, OR, USA) with 24 cameras using a sampling frequency of 100 Hz. Fifty-seven markers were attached in accordance with the Biomech-57 marker set^32^. Four extra markers were attached to the floor to capture the floor motion. Ground reaction force and moment were collected using the AMTI force plates system (AMTI, Advanced Mechanical Technology Inc., size 120 cm by 120 cm), sampled at 1 kHz. The raw marker trajectories and force plate data were digitally filtered using a fourth-order, 10 Hz low-pass Butterworth filter. Force plate data at 1 kHz were down-sampled to a common sample rate of 100 Hz by using a spline method.

All the data were processed in MATLAB 2022b (MathWorks, Inc., Natick, Massachusetts). The 3D kinematics were computed using adjacent segments for the ankle (foot to shank), knee (shank to thigh), hip (thigh to pelvis), and torso (pelvis to torso). The method of modelling was based on Euler with X-Y-Z series of rotation as per Robertson et al. (2013)^33^ and HAS Motion (2024)^34^. Discrete variables, including the joint angle range of motion and the root mean square (RMS) of angular velocity, were computed. The angular velocity of the joints was determined by taking the second derivative of the joint angle. The COP and Time-to-Boundary in the mediolateral direction (TTB-ML)^26^ measures were calculated, including standard deviation of sway amplitude (SDAmp; i.e. SD of COP amplitude from the centre of the trajectory) and sway velocity^35^. The markers placed on the first, fifth metatarsal joints, the first distal phalanx and the calcaneus were defined as borders of the foot, which is modelled as a rectangle for TTB calculations (Supplementary Figure S1).

### Principal component analysis

In the present study, principal component analysis (PCA) aimed to analyse the joint kinematic patterns during one-leg stance that are different between CAI from HC. The PCA is a multivariate statistical method that analyses data waveforms by focusing on their variance. PCA produces principal component vectors (PCVs) and associated scores (PCSs) for each PCV. The PCVs capture the axes of variance, and the PCSs represent the projections of original data onto each PCV. If the PCSs for CAI significantly differ from those for HC on any PCV, the joint kinematic features linked to that PCV can be considered key patterns for differentiating two groups.

In this study, the joint angle average was used for the PCA which was followed by six steps. First, the intra-participant average was calculated for each time point within the three sets of data obtained from each participant. Second, mean centring was conducted on each of 12,000 variables (i.e., averages for 1,000 time points, four angles in three planes) using the z-score:

$${{\text{Z}}_{\text{t}}}=({{\text{X}}_{\text{t}}} - {{{\upmu }}_{\text{t}}})/{{\text{s}}_{\text{t}}},$$

where Z_t_ is the z-score for the parameter t, X_t_ is the raw data of the parameter t, µ_t_ is the mean of the parameter t for the participant, and σ_t_ is the standard deviation of the parameter t. Third, input matrices of 46 participants by 12,000 variables were constructed. Fourth, principal component vectors were extracted until the cumulate description attained 90% of the total variance. Fifth, statistical analyses were conducted to identify the main effects of CAI on the joint kinematic patterns represented by the principal component vectors. Finally, for each principal component vector, simulated kinematic waveforms were reconstructed from the principal component scores with mean scores across groups, to interpret data on the average joint angle corresponding to the principal component vectors. The leave-one-out cross-validation technique was conducted to validate the results of the main PCA and confirm the stability of the techniques, by performing 46 surrogate PCAs^36^. The dimensions of the surrogate input matrix were 45 by 12,000.

### Statistics

To identify differences in balance between subjects with CAI and HC under two balance conditions, a two-way repeated ANOVA (2 groups × 2 conditions) was applied to TTB-ML mean of minima, TTB-ML absolute of minima, TTB-ML SD of minima, once the normal distribution of data was confirmed. A Bonferroni post hoc test was computed when significant interactions were observed. Partial Eta Squared effect sizes were used to determine the magnitude of differences and were interpreted as small (0.01–0.06), moderate (> 0.06–0.14), and large (> 0.14)^37^. The joint kinematic patterns during static and moving conditions were analysed by PCA. To determine the PCVs that differentiate from CAI to HC, independent sample t-tests were conducted on the PCSs of each PCV between groups, similar to the methods used in the previous study^21^. Cohen’s d effect size was calculated to validate the t-test results, with interpretation as small (< 0.40), moderate (0.40–0.80), and large (> 0.80)^38^. To explore associations between the kinematics pattern with balance control, Pearson’s correlation analysis examined the relationship between the PCS of significant PCV with TTB-ML measures, standard deviation of sway amplitude (SDAmp) and the sway velocity of COP. To link kinematics with balance control observed, a stepwise multiple regression was conducted to identify which joint angular velocities and in which specific planes (sagittal, frontal, and horizontal) at the ankle, knee, hip, and torso significantly contribute to TTB-ML mean of minima. The statistical significance was set at *p* < 0.05.

## Results

A significant interaction was observed for all three TTB-ML measures (mean of minima: *p* = 0.001, *ηp*^*2*^ = 0.211; absolute of minima: *p* = 0.016, *ηp*^*2*^ = 0.125; SD of minima: *p* = 0.003, *ηp*^*2*^ = 0.183). Significant group main effects were also observed and pairwise comparisons revealed that the CAI group on average demonstrated significantly lower TTB-ML in all three measures (Table 2). Post hoc analysis revealed that the CAI group displayed significantly lower TTB-measures during static conditions compared to the control group (mean of minima: *p* = 0.001, *ηp*^*2*^ = 0.208; absolute of minima: *p* = 0.011, *ηp*^*2*^ = 0.139; SD of minima: *p* = 0.005, *ηp*^*2*^ = 0.168), but no significant between-group difference during moving condition. Significant condition main effects were observed, and post hoc analysis demonstrated the significantly decreased TTB measures in both groups in the moving platform condition compared to static (Table 2).

The PCA for moving conditions revealed that the first 10 PCVs explained > 90% of the joint movement patterns (Supplementary Table S1) during a 10-second balance on the moving platform. Among these 10 PCVs, the PCS of PCV3 (*p* < 0.05, ES =-0.62) and PCV7 (*p* < 0.05, ES =-0.61) revealed significant differences in the joint angles between CAI and HC. The results are consistent with those for the surrogate PCAs, revealing either significant differences (37 of the 46 surrogate PCAs) or marginally significant differences (9 of 46 surrogate PCAs) (Supplementary Table S2). The PCA for static conditions revealed that the first 8 PCVs explained > 90% of the joint movement patterns (Supplementary Table S3) during a 10-second balance on the static platform, validated by leave-one-out cross-validation (Supplementary Table S4). PCS of PCV2 (*p* < 0.05, ES = 0.69) revealed significant differences in joint angles between groups.

**Fig. 2:**
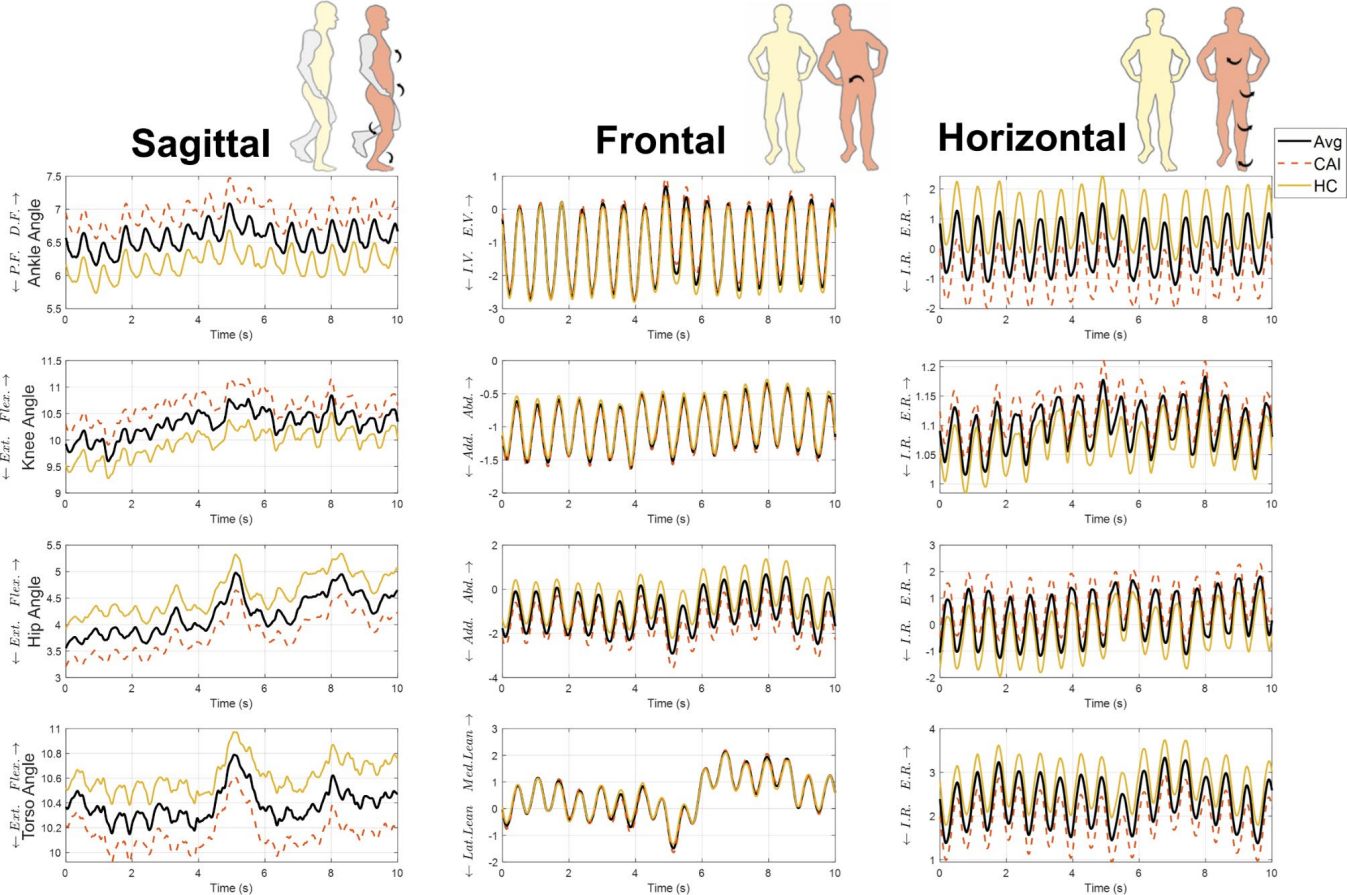
Joint kinematics reconstructed from the PCS of PCV3. Posture diagram: the right panel of each plane illustrated a specific movement pattern in CAI, with arrows indicating the kinematic differences compared to HC by PVC3. The waveforms: were reconstructed by the mean of PCS in participants with CAI, HC and all participants of PCV3. Avg. average of all participants. The plots show ankle, knee, hip and torso angles across sagittal, frontal, and horizontal planes over 10 s. The definitions of the abbreviations in the graph are as follows: D.F. dorsiflexion, P.F. plantarflexion, Flex. flexion, Ext. extension, E.V.: Eversion, I.V.: Inversion, Add. adduction, Abd. abduction, Lat. lateral, Med. Medial, I.R. internal rotation, E.R. external rotation. The figure was plotted by Matlab 2022b (https://uk.mathworks.com/products/matlab.html).

**Fig. 3:**
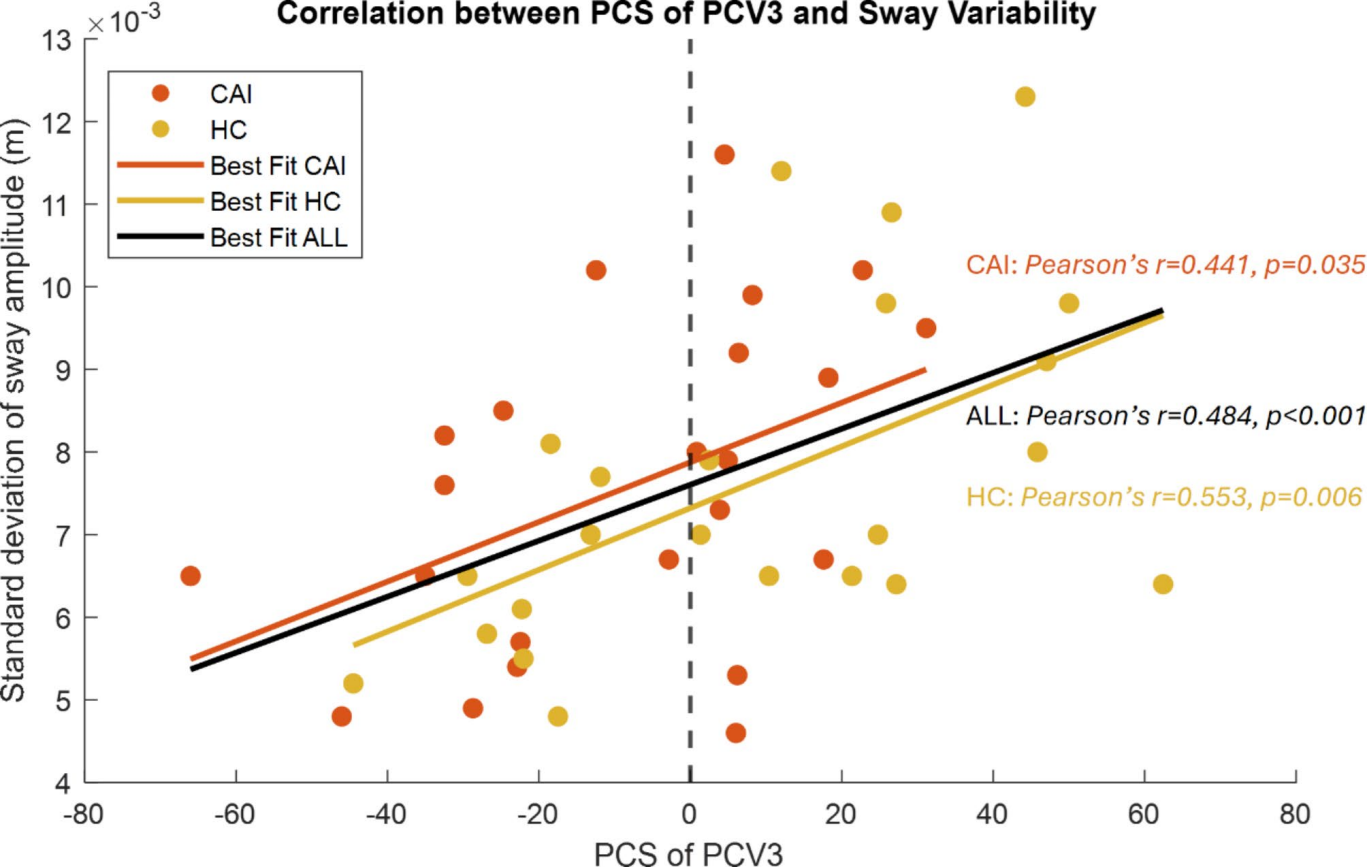
The correlation between the principal component score (PCS of PCV3) and the standard deviation of sway amplitude of the centre of pressure. The scatter plot displays positive correlations as indicated by the best-fit lines for CAI, HC and combined data (ALL). Both groups and the combined data showed statistically significant correlations.

Since the PCSs for the two vectors were both negative on average for the CAI group and positive for the HC, and the PCV3 explained a larger variance of movement, the reconstructed waveforms with the mean score of each group can be interpreted as characteristics. Figure 2 shows the recombined joint angles position of ankle, knee, hip, and torso across sagittal, frontal, and horizontal planes for PCV3 during moving conditions. As can be seen from Fig. 2, CAIs exhibited a more dorsiflexed ankle, more flexed knee, less flexed hip and torso, hip tended to be more adducted, ankle more internally rotated, knee and hip more externally rotated, and torso less externally rotated. Pearson’s correlation revealed a significant positive relationship between PCS of PCV3 and SDAmp in the combined data (*r* = 0.484, *p* < 0.001) and in both CAI and HC groups individually (Fig. 3), suggesting that HC movement strategies, characterised by positive PCS scores, involve greater COP amplitude variability. Similarly, PCS of PCV3 was significantly correlated with sway velocity in the combined data (*r* = 0.332, *p* < 0.05) and the CAI group (*r* = 0.609, *p* = 0.002), but not in the HC group (*r* = 0.155, *p* = 0.48). No associations were found between the PCS of PCV3 and TTB-ML measures (*p* > 0.1). The reconstructed PCV2 for the static condition (Supplementary Figure S2) indicates that CAI participants exhibited similar ankle dorsiflexion to HC but with less flexed knee and hip. CAI was characterized by increased ankle inversion, knee adduction, and internal ankle rotation, along with greater external hip rotation. No associations were found between PCS of PCV2 and SDAmp (*r* = 0.11 *p* > 0.1), sway velocity (*r*=-0.016 *p* > 0.5), or TTB-ML measures (*p* > 0.1).

**Fig. 4:**
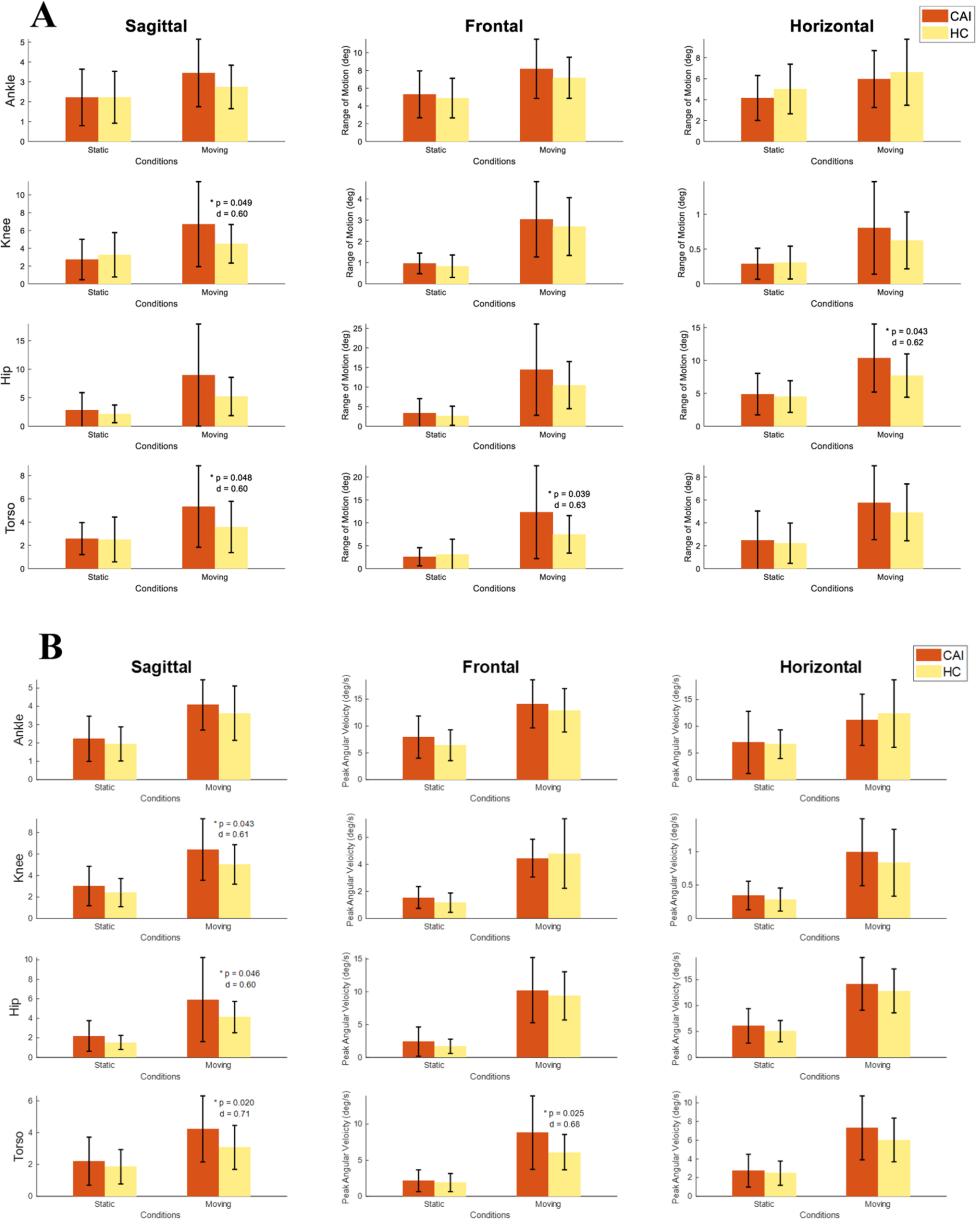
**A** revealed the range of motion (joint angle) during 10 s balance on both static and moving platform condition. The ranges of motion were calculated as the maximum angle minus the minimum angle. **B** The angular velocity was calculated as the time-derivative of joint angle. The root mean squared angular velocity. Data are expressed as means ± SD. The data were analysed using independent t-tests for comparisons between CAI and HC groups. The “*” symbol indicates significant differences between the two groups (*p < 0.05). P-values and effect sizes (Cohen’s d) are shown.

Compared with the original data in the range of motion (ROM, max angle minus minimum angle), and RMS angular velocity during a 10-s moving platform, the CAI group revealed a significantly greater ROM and higher RMS angular velocity in the knee, hip, and torso (Fig. 4).

As per Tables 3 42.1% of the variance in TTB-ML mean of minima could be explained by ankle angular velocity in the sagittal and frontal planes, and hip angular velocity in the horizontal plane (F _(3,43)_ = 10.186, *p* < 0.001). The final regression model revealed that higher ankle velocities in the sagittal and frontal planes were correlated with shorter TTB-ML mean of minima, indicating poorer stability; while higher hip velocities in the horizontal plane correlated with greater TTB-ML, indicating better stability and accounting for an additional 8.7% of the variance.

Results Tables and Figures.

**Table 2:** Time to boundary (TTB) measures (Mean ± SD) for the CAI and HC on static and moving balance condition.

	Group								*P* values (partial Eta squared)				95% CI of the difference			
	CAI				HC				Group		Condition		Group		Condition	
	Static		Moving		Static		Moving		*p*	ηp^2^	*p*	ηp^2^	(Lower, upper)		(Lower, upper)	
TTB ML mean minimum (s)	1.14^†^	± 0.45	0.44^‡^	± 0.12	1.73	± 0.70	0.48^‡^	± 0.19	0.003	(0.19)	< 0.001	(0.77)	-0.52,	-0.12	0.81,	1.13
TTB ML absolute minimum (s)	0.12^†^	± 0.07	0.05^‡^	± 0.03	0.20	± 0.14	0.06^‡^	± 0.04	0.013	(0.13)	< 0.001	(0.51)	-0.09,	-0.01	0.07,	0.14
TTB ML SD minimum (s)	1.20^†^	± 0.49	0.47^‡^	± 0.13	1.81	± 0.85	0.49^‡^	± 0.21	0.01	(0.14)	< 0.001	(0.73)	-0.55,	-0.08	0.84,	1.22

† Denotes significant difference from the control group (*p* < 0.05) within the same condition. Bonferroni adjusted for multiple comparisons.

‡ Denotes significant difference than the static balance condition (*p* < 0.05) within the group. Bonferroni adjusted for multiple comparisons.

Effect Sizes were estimated by partial Eta squared (ηp^2^). CI, confidence interval.

**Table 3:** Stepwise multiple liner regression analysis root mean squared velocity predicting TTBML mean of minima.

Dependent variable	Independent variables	*R*-squared	Coefficients (β) [95% CI]	*p*-Values
TTB ML mean of minima	RmsVAnkleSag.	0.251	-5.460 [-8.635, -2.284]	< 0.001
	RmsVAnkleFro.	0.334	-2.375 [-3.744, -1.005]	< 0.001
	RmsVHipHor.	0.421	1.607 [0.321, 2.892]	0.016

The definitions of the abbreviations in the graph are as follows: RmsVAnkleSag.: The RMS angular velocity of ankle in sagittal plane; RmsVAnkleFro.: The RMS angular velocity of ankle in frontal plane; RmsVHipHor.: The RMS angular velocity of hip in horizontal plane.

## Discussion

The study applied a challenging balance task, aiming to identify differences in balance and relevant movement strategies between people with CAI and healthy control, as well as identify the underlying movement strategies that contribute to balance control. Partially confirming our hypothesis, the CAI group exhibited significantly lower TTB measures of balance during the static condition, although no significant differences were observed between CAI and HC groups during the moving condition. The CAI group exhibited a specific balance strategy whilst the platform was moving, characterised by a more dorsiflexed ankle, more flexed knee, less flexed hip and torso, more adducted hip, more internally rotated ankle, more externally rotated knee and hip, and less externally rotated torso. The CAI had a significantly greater range of motion and higher angular velocity in the knee, hip, and torso compared to HC during the moving condition.

The present findings align with previous research on static one-leg stance, demonstrating lower TTB in the CAI group compared to healthy controls, as documented by Hertel and Olmsted-Kramer (2007)^26^ and Wikstrom et al. (2010)^39^. However, discrepancies emerge in more demanding weight-bearing tasks. Knapp (2011)^40^ and Song et al. (2017)^41^ observed no significant differences in balance control under eyes-closed standing conditions, in contrast to McKeon and Hertel (2008)^42^ findings of a reduced TTB in the CAI. Furthermore, during dynamic balance tasks, such as lateral stepping down, individuals with CAI exhibited a lower TTB^9^, highlighting varied responses in balance control under different testing conditions.

The absence of differences in TTB during moving platform tasks in the current study might be explained by hip compensation. As demonstrated by our regression model, higher ankle velocities in the sagittal and frontal planes correlated with lower TTB ML, suggesting poorer stability, while higher hip velocities in the horizontal plane correlated with higher TTB ML, suggesting better stability. The enhanced hip compensation in CAI (Fig. 4b), not only improves balance but also mitigates fast ankle movement, which is particularly relevant to ankle sprain^43,44^. This overcompensation could obscure group differences, potentially causing floor or ceiling effects that make balance differences undetectable. Additionally, the use of a moving platform may enhance muscle co-contraction and ankle stiffness, potentially masking balance differences between groups. During quiet standing with low postural demands, intrinsic stiffness provides most of the stability^45^, while dynamic conditions require sufficient co-contraction to counteract destabilising perturbations^46,47^. Muscle co-contraction is modulated through feedforward mechanisms^48^, with the central nervous system adjusting postural strategies based on experience^49^. In this study, individuals with CAI may have adopted a co-contracted strategy, with repetitions and practice enabling feedforward adjustments that reduced group differences over time. While this study cannot provide direct evidence of muscle co-contraction, future research should investigate its role in balance control, particularly during sinusoidal perturbations that challenge both agonist and antagonist. Additionally, perturbation tasks should be carefully designed to avoid excessive difficulty, minimising potential practice effects and feedforward control adjustments.

To further understand the balance strategies applied by individuals with CAI, the present findings of greater ROM and angular velocity in the lower limb and torso, suggest enhanced whole-body motor compensation. It has been observed that CAI attempted to adapt their movement patterns from ankle- to hip-based strategies to compensate for partially deafferented ankle joints to maintain postural control^50^. In CAI, increased ROM and angular velocity in the knee, hip and torso, suggest the utilization of these joints to adjust the postural control in response to the moving base of support, thus compensating for the avoidance of excessive ankle movements. Additionally, the increase in ROM and angular velocity in the torso might reveal an inability to stabilise the trunk effectively, which is in line with a study by McCann et al. (2021)^16^, who reported a decrease in contractility of the transversus abdominis in CAI. The transversus abdominis provides trunk dynamic stability by absorbing the energy of loading the lumbar spine during activities^51^. The orientation and control of the COM tends to be managed more proximally by the hip, a strategy that may enhance stability, as evidenced by our regression model, highlighting the potential importance of COM positioning during balance tasks to be explored in future work.

In addition to identifying compensatory movements through discrete kinematics, this study explored specific movement patterns in CAI by using PCA, providing insights that enable rehabilitation to target not only the injured area but also the functional integration of the kinetic chain for effective recovery and injury prevention. The PCA identified distinct whole-body movement strategies in individuals with CAI under both static and moving conditions, but the difference of kinematic pattern is task-specific, varying between static and dynamic conditions. CAI participants showed greater ankle inversion in static conditions, but this difference diminished in dynamic tasks, likely due to the platform generating comparable mediolateral forces, resulting in similar frontal plane movement patterns between groups. During moving conditions, CAI showed greater ankle dorsiflexion compared to HC than in static conditions, suggesting a strategy of foot dorsiflexion coupled with calcaneal dorsiflexion and eversion to limit excessive ankle inversion^52,53^. Moreover, CAIs exhibited a greater ankle internal rotation under both conditions, consistent with previous findings on cutting manoeuvres in individuals with CAI^54^, and lab-captured episodes of ‘giving way’^55–57^. This increased ankle internal rotation observed during closed-chain tasks in this study likely reflects an externally rotated tibia, attributed to laxity in the anterior talofibular ligament that provide static stability to the talocrural joint, leading to greater tibial external rotation. An increase in hip external rotation in CAI in the present results, in line with a landing study^58^, might be due to the coupling between ankle internal rotation and hip external rotation as evidenced by Souza et al. (2010)^59^.

Together with greater joint ROM, angular velocity indicating challenges in stabilising both distal and proximal joints, and PCA findings revealing an altered kinetic chain in CAI, collectively describe the phenomenon – altered movement strategies. The alterations in the kinetic chain might further contribute to other musculoskeletal conditions, such as low back pain. It should be noted that the low explained variance by PCA in PCV2 (17%) during static and PCV3 (13%) during dynamic conditions suggests that kinetic chain alterations may be individual, requiring further investigation to confirm. These alterations likely stem from differences in musculoskeletal structures (e.g., muscles, fascia, ligaments, tendons), injury history, physical activity, sport demands, and psychological factors^4^. Therefore, future research should identify CAI-specific kinematic patterns, understand the mechanisms underlying these patterns, and develop physiotherapy interventions to restore proper movement patterns. Rehabilitation programs for CAI should aim to restore the proper kinetic chain by addressing not only the injured area but also the integration of the entire musculoskeletal system, supporting effective recovery and helping prevent secondary injuries. For example, applying biofeedback to improve body alignment during functional exercise^60,61^. However, the exclusion of CAI copers from this study limits our understanding of the full spectrum of those who have sprained their ankles but have no residual symptoms, particularly whether improved ankle function restores postural compensation. If improved ankle function allows for a postural strategy similar to healthy individuals, this would highlight the importance of addressing the entire kinetic chain in rehabilitation.

A significant positive correlation was observed between the PCS of PCV3 and both sway variability and sway velocity, while no correlation was found between the PCS of PCV2 during static standing, suggesting that kinematic differences during static conditions are unrelated to postural control but become more pronounced as postural demands increase. Specifically, the movement strategies employed by HC, characterized by positive PCS scores, involve higher COP sway velocity and greater variability in COP amplitude under high-demand balance conditions. An increase in sway velocity and variability does not necessarily reflect deficits of dynamic balance. For instance, higher COP velocities have been observed in healthy control participants compared to an anterior-cruciate ligament deficient group during a static one-leg stance, indicating the importance of normal active sway in finding a stable solution to postural challenges^62^. Additionally, higher COP velocities could indicate exploratory behaviours in seeking stable performance solutions under novel task constraints. This is evidenced by significantly increased COP velocities under biofeedback conditions during bipedal stance on a stable and unstable platform compared to a condition without biofeedback^63^. The observed increase in variability and velocity in the present study may also relate to error-based learning. Such that, motor variability, actively regulated by our neuromuscular system, proves more efficient in facilitating motor learning than reward-based regulation^64^. Although not straightforward, it is plausible that a decrease in the duration between consecutive COP positions might reflect an enhanced ability of muscles to adjust their length and counteract body sway. Therefore, we suggest that the relevance of COP motion might be considered in line with COM motion, thus providing a link between the strategy and the performance.

There are several limitations in the study that must be addressed. Firstly, we did not account for variations in CAI severity, which might influence the results^65^. CAIT measures self-perceived ankle instability, including psychological factors such as kinesiophobia, which may have influenced self-organization strategies and balance performance^4^. The CAIT score of our CAI participants, 17.3 ± 3.5, aligns with those reported in previous studies^9,54^, suggesting that our findings may only apply to individuals with similar ankle function. We observed that participants with CAI took longer and required more repetitions to achieve three successful balances, suggesting that practice might have enabled feedforward adjustments and increased muscle co-contraction to a sufficient level^49^, reducing group differences over time. However, we did not quantify repetitions, which may have influenced the results. Future studies should record the number of attempts during challenging tasks to evaluate motor adaptation or develop valid measures for failed balance trials to better track progression. Finally, requiring participants to keep their hands on their waists excluded arm movements, limiting the applicability of our findings to real-world scenarios where arms are actively used for balance.

## Conclusion

Individuals with CAI displayed differences in balance to healthy controls during a one-leg stance on the static platform, but not on the moving platform. On the moving platform, CAI participants demonstrated greater proximal compensations, with significantly increased ROM and angular velocity in the knee, hip and torso. These findings suggest an altered movement strategy in CAI, where ankle injuries impair the ability to stabilize both distal and proximal joints, and an altered kinetic chain from ankle to torso. Future research should track motor adaptation during challenging tasks, particularly those prone to failure, to better understand these mechanisms. Additionally, studies should identify CAI-specific kinematic patterns, explore their relation to ankle function, and include CAI COPERs to determine if improved ankle function enables postural strategies similar to healthy individuals. Addressing the kinetic chain in rehabilitation may enhance recovery and prevent secondary injuries.

## Electronic supplementary material

Below is the link to the electronic supplementary material.

Supplementary Material 1

## Data Availability

The datasets analysed during the current study are available from the corresponding author (XX) on reasonable request.
