# Supplementary material for: Kinematics of balance controls in people with chronic ankle instability during unilateral stance on a moving platform
Source: Sci Rep. 2025 Jan 7;15:1126. doi: 10.1038/s41598-025-85220-x (PMC11707225; doi:10.1038/s41598-025-85220-x)
Supplement: Supplementary file 1 — Supplementary Material 1 [file 41598_2025_85220_MOESM1_ESM.docx]

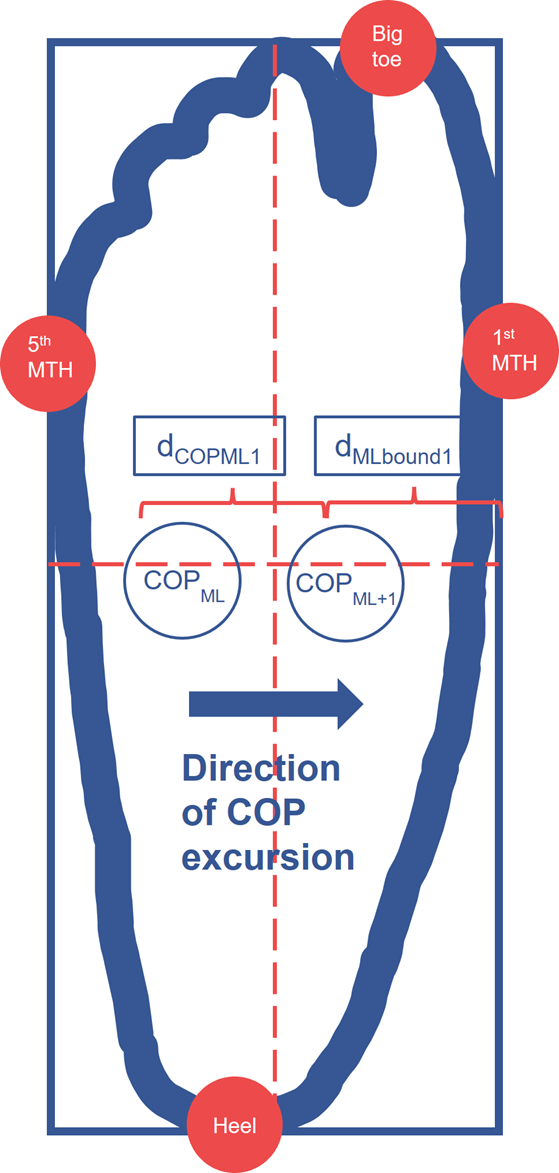


Supplementary Figure S1. The rectangle is modelled using the vector from the 5^th^ (5^th^ MTH) to 1^st^ (1^st^ MTH) metatarsal heads as the width (X-axis) and an orthogonal vector as the height (Y-axis), with the height length determined by projecting the vector from the calcaneus (heel) to the first distal phalanx (near the end of big toe) onto the Y-axis. The COP is transformed from the global coordinate system to the local rectangle system by translating the COP relative to the heel and projecting it onto the rectangle's X and Y axes. TTB is calculated based on the distance between the local COP and the rectangle boundary in the local mediolateral (TTB ML) direction, divided by the COP velocity (calculated as the change in the COP's position along the X-axis between consecutive time steps divided by the time interval) in the respective direction.

Supplementary Table S1. Results of main PCA during moving condition. The main PCA generated a total of 10 PCVs to retain 90% of the variance. The PCV3 and PCV7 revealed significant differences between the CAI and HC

|  | PCV1 | PCV2 | PCV3 | PCV4 | PCV5 | PCV6 | PCV7 | PCV8 | PCV9 | PCV10 |
| --- | --- | --- | --- | --- | --- | --- | --- | --- | --- | --- |
| Explained variance (%) | 22.730 | 14.997 | 13.365 | 11.660 | 8.106 | 5.979 | 4.563 | 3.383 | 3.182 | 2.271 |
| Cumulative (%) | 22.730 | 37.728 | 51.093 | 62.753 | 70.859 | 76.838 | 81.401 | 84.783 | 87.966 | 90.237 |
| CAI: mean (SD) | -0.801 | 1.224 | -8.495 | 5.152 | -2.262 | 0.441 | -4.869 | 1.838 | 2.824 | -1.223 |
|  | ±39.061 | ±28.121 | ±24.451 | ±24.072 | ±20.961 | ±19.647 | ±15.117 | ±17.995 | ±14.703 | ±13.947 |
| HC: mean (SD) | 0.801 | -1.224 | 8.495 | -5.152 | 2.262 | -0.441 | 4.869 | -1.838 | -2.824 | 1.223 |
|  | ±35.917 | ±32.631 | ±30.127 | ±28.463 | ±23.548 | ±18.830 | ±16.954 | ±9.398 | ±12.708 | ±9.155 |
| *P* value on PC scores | 0.886 | 0.787 | 0.041^*^ | 0.192 | 0.495 | 0.877 | 0.046^*^ | 0.390 | 0.170 | 0.486 |
| Cohen's-d | -0.043 | 0.08 | -0.619 | 0.391 | -0.203 | 0.046 | -0.606 | 0.256 | 0.411 | -0.207 |

^*^ *p* < 0.05

Supplementary Table S2. Results of the surrogate PCAs during moving conditions. The significant differences between CAI and HC were generated from the 46 surrogate PCAs regarding the PCSs of the PCVs.

|  | sPCA1 | sPCA2 | sPCA3 | sPCA4 | sPCA5 | sPCA6 | sPCA7 | sPCA8 | sPCA9 | sPCA10 | sPCA11 | sPCA12 | sPCA13 |
| --- | --- | --- | --- | --- | --- | --- | --- | --- | --- | --- | --- | --- | --- |
| PCV revealed significance | PCV 7 | PCV 3 | PCV 3 | PCV 3 | PCV 3 | PCV 3 | PCV 3 | PCV 3 | PCV 3 | PCV 3 | PCV 3 | PCV 3 | PCV 7 |
| p value | 0.038^†^ | 0.023^†^ | 0.037^†^ | 0.043^†^ | 0.041^†^ | 0.036^†^ | 0.025^†^ | 0.044^†^ | 0.027^†^ | 0.040^†^ | 0.041^†^ | 0.060^‡^ | 0.074^‡^ |
| Cohen's-d | -0.456 | -0.504 | -0.458 | -0.445 | -0.448 | -0.461 | 0.494 | -0.441 | 0.489 | -0.451 | -0.450 | -0.411 | -0.391 |
|  | sPCA14 | sPCA15 | sPCA16 | sPCA17 | sPCA18 | sPCA19 | sPCA20 | sPCA21 | sPCA22 | sPCA23 | sPCA24 | sPCA25 | sPCA26 |
| PCV revealed significance | PCV 7 | PCV 3 | PCV 3 | PCV 7 | PCV 3 | PCV 3 | PCV 3 | PCV 3 | PCV 3 | PCV 3 | PCV 7 | PCV 3 | PCV 3 |
| p value | 0.027^†^ | 0.015^†^ | 0.041^†^ | 0.041^†^ | 0.033^†^ | 0.032^†^ | 0.080^‡^ | 0.041^†^ | 0.042^†^ | 0.045^†^ | 0.058^‡^ | 0.019^†^ | 0.023^†^ |
| Cohen's-d | -0.488 | -0.539 | -0.449 | -0.449 | -0.469 | -0.472 | 0.383 | -0.449 | -0.447 | -0.440 | -0.415 | -0.522 | -0.503 |
|  | sPCA27 | sPCA28 | sPCA29 | sPCA30 | sPCA31 | sPCA32 | sPCA33 | sPCA34 | sPCA35 | sPCA36 | sPCA37 | sPCA38 | sPCA39 |
| PCV revealed significance | PCV 3 | PCV 3 | PCV 7 | PCV 7 | PCV 7 | PCV 3 | PCV 3 | PCV 3 | PCV 3 | PCV 3 | PCV 3 | PCV 3 | PCV 3 |
| p value | 0.043^†^ | 0.018^†^ | 0.044^†^ | 0.049^†^ | 0.017^†^ | 0.031^†^ | 0.029^†^ | 0.029^†^ | 0.033^†^ | 0.024^†^ | 0.016^†^ | 0.027^†^ | 0.054^‡^ |
| Cohen's-d | -0.445 | -0.525 | -0.443 | -0.431 | -0.532 | -0.476 | -0.483 | -0.482 | -0.469 | -0.500 | -0.532 | -0.489 | -0.422 |
|  | sPCA40 | sPCA41 | sPCA42 | sPCA43 | sPCA44 | sPCA45 | sPCA46 |  |  |  |  |  |  |
| PCV revealed significance | PCV 3 | PCV 3 | PCV 8 | PCV 3 | PCV 3 | PCV 3 | PCV 3 |  |  |  |  |  |  |
| p value | 0.093^‡^ | 0.098^‡^ | 0.044^†^ | 0.035^†^ | 0.045^†^ | 0.054^‡^ | 0.059^‡^ |  |  |  |  |  |  |
| Cohen's-d | 0.366 | 0.361 | -0.443 | -0.464 | 0.441 | 0.423 | -0.413 |  |  |  |  |  |  |
| † *p*<0.05. ‡ *p*<0.1 |  |  |  |  |  |  |  |  |  |  |  |  |  |


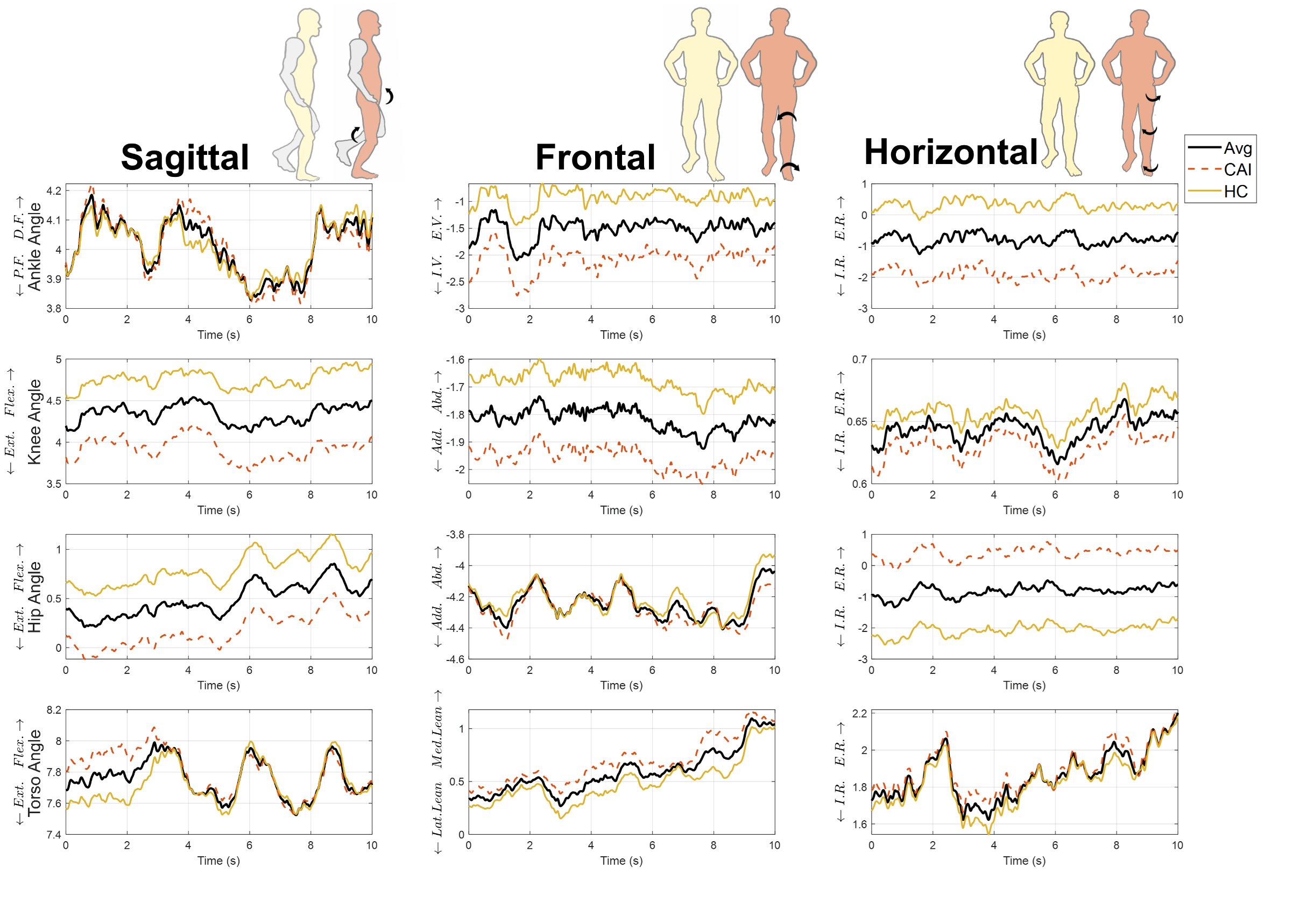


Supplementary Figure S2. Joint kinematics reconstructed from the PCS of PCV2. Posture diagram: the right panel of each plane illustrated a specific movement pattern in CAI, with arrows indicating the kinematic differences compared to HC by PVC2. The waveforms: were reconstructed by the mean of PCS in participants with CAI, HC and all participants of PCV2. Avg. average of all participants. The plots show ankle, knee, hip and torso angles across sagittal, frontal, and horizontal planes over 10 seconds. The definitions of the abbreviations in the graph are as follows: D.F. dorsiflexion, P.F. plantarflexion, Flex. flexion, Ext. extension, E.V.: Eversion, I.V.: Inversion, Add. adduction, Abd. abduction, Lat. lateral, Med. Medial, I.R. internal rotation, E.R. external rotation. The figure was plotted by Matlab 2022b (https://uk.mathworks.com/products/matlab.html)

Supplementary Table S3. Results of main PCA during static condition. The main PCA generated a total of 8 PCVs to retain 90% of the variance. The PCV2 revealed significant differences between the CAI and HC.

|  | PCV1 | PCV2 | PCV3 | PCV4 | PCV5 | PCV6 | PCV7 | PCV8 |
| --- | --- | --- | --- | --- | --- | --- | --- | --- |
| Explained variance (%) | 29.347 | 17.306 | 12.574 | 11.405 | 9.135 | 5.873 | 3.888 | 2.821 |
| Cumulative (%) | 29.347 | 46.653 | 59.227 | 70.632 | 79.767 | 85.640 | 89.528 | 92.349 |
| CAI: mean (SD) | -3.854 | 12.067 | 0.746 | 4.325 | -0.466 | 0.942 | 1.523 | 1.949 |
|  | ±46.273 | ±34.058 | ±34.414 | ±31.377 | ±25.110 | ±22.071 | ±15.618 | ±15.918 |
| HC: mean (SD) | 3.854 | -12.067 | -0.746 | -4.325 | 0.466 | -0.942 | -1.523 | -1.949 |
|  | ±50.237 | ±36.122 | ±28.750 | ±28.307 | ±28.824 | ±21.231 | ±19.321 | ±13.784 |
| p value on PC scores | 0.591 | 0.024* | 0.874 | 0.332 | 0.907 | 0.769 | 0.559 | 0.379 |
| Cohen's-d | -0.16 | 0.687 | 0.047 | 0.289 | -0.034 | 0.087 | 0.173 | 0.262 |

^*^ *p* < 0.05

Supplementary Table S4. Results of the surrogate PCAs during static conditions. The significant differences between CAI and HC were generated from the 46 surrogate PCAs regarding the PCSs of the PCVs.

|  | sPCA1 | sPCA2 | sPCA3 | sPCA4 | sPCA5 | sPCA6 | sPCA7 | sPCA8 | sPCA9 | sPCA10 | sPCA11 | sPCA12 | sPCA13 |
| --- | --- | --- | --- | --- | --- | --- | --- | --- | --- | --- | --- | --- | --- |
| PCV revealed significance | PCV 2 | PCV 2 | PCV 2 | PCV 2 | PCV 2 | PCV 2 | PCV 2 | PCV 2 | PCV 2 | PCV 2 | PCV 2 | PCV 2 | PCV 2 |
| p value | 0.037^†^ | 0.026^†^ | 0.044^†^ | 0.057‡ | 0.020^†^ | 0.030^†^ | 0.019^†^ | 0.018^†^ | 0.018^†^ | 0.037^†^ | 0.017^†^ | 0.032^†^ | 0.034^†^ |
| Cohen's-d | 0.460 | 0.492 | 0.443 | 0.417 | 0.517 | 0.480 | 0.519 | 0.522 | 0.525 | 0.459 | 0.530 | 0.472 | 0.467 |
|  | sPCA14 | sPCA15 | sPCA16 | sPCA17 | sPCA18 | sPCA19 | sPCA20 | sPCA21 | sPCA22 | sPCA23 | sPCA24 | sPCA25 | sPCA26 |
| PCV revealed significance | PCV 2 | PCV 2 | PCV 2 | PCV 2 | PCV 2 | PCV 2 | PCV 2 | PCV 2 | PCV 2 | PCV 2 | PCV 2 | PCV 2 | PCV 2 |
| p value | 0.033^†^ | 0.019^†^ | 0.025^†^ | 0.033^†^ | 0.040^†^ | 0.016^†^ | 0.027^†^ | 0.021^†^ | 0.016^†^ | 0.027^†^ | 0.041^†^ | 0.024^†^ | 0.029^†^ |
| Cohen's-d | 0.471 | 0.520 | 0.496 | 0.469 | -0.452 | 0.534 | 0.487 | 0.509 | 0.534 | 0.488 | 0.449 | 0.499 | 0.482 |
|  | sPCA27 | sPCA28 | sPCA29 | sPCA30 | sPCA31 | sPCA32 | sPCA33 | sPCA34 | sPCA35 | sPCA36 | sPCA37 | sPCA38 | sPCA39 |
| PCV revealed significance | PCV 2 | PCV 2 | PCV 2 | PCV 2 | PCV 2 | PCV 2 | PCV 2 | PCV 2 | PCV 2 | PCV 2 | PCV 2 | PCV 2 | PCV 2 |
| p value | 0.015^†^ | 0.019^†^ | 0.049^†^ | 0.040^†^ | 0.036^†^ | 0.013^†^ | 0.030^†^ | 0.027^†^ | 0.011^†^ | 0.032^†^ | 0.022^†^ | 0.027^†^ | 0.029^†^ |
| Cohen's-d | 0.542 | 0.522 | 0.432 | 0.452 | 0.462 | 0.555 | 0.480 | 0.489 | 0.565 | 0.473 | 0.505 | 0.489 | 0.480 |
|  | sPCA40 | sPCA41 | sPCA42 | sPCA43 | sPCA44 | sPCA45 | sPCA46 |  |  |  |  |  |  |
| PCV revealed significance | PCV 2 | PCV 2 | PCV 2 | PCV 2 | PCV 2 | PCV 2 | PCV 2 |  |  |  |  |  |  |
| p value | 0.037^†^ | 0.028^†^ | 0.017^†^ | 0.016^†^ | 0.023^†^ | 0.043^†^ | 0.029^†^ |  |  |  |  |  |  |
| Cohen's-d | 0.460 | 0.483 | 0.532 | 0.532 | 0.502 | 0.444 | 0.483 |  |  |  |  |  |  |
| † *p*<0.05. ‡ *p*<0.1 |  |  |  |  |  |  |  |  |  |  |  |  |  |
